# Supplementary material for: Combined chemical genetics and data-driven bioinformatics approach identifies receptor tyrosine kinase inhibitors as host-directed antimicrobials
Source: Nat Commun. 2018 Jan 24;9:358. doi: 10.1038/s41467-017-02777-6 (PMC5783939; doi:10.1038/s41467-017-02777-6)
Supplement: Supplementary file 2 — Supplementary Information [file 41467_2017_2777_MOESM2_ESM.pdf]

Supplementary Methods

A flow cytometry-based readout for intracellular bacterial load.

To uncover host pathways controlling intracellular bacterial survival, we developed a fast, robust and novel assay suitable for medium-throughput (96-well) compound and siRNA screening, employing flow cytometry as a readout for intracellular bacterial load using fluorescent strains of *Stm* and *Mtb.* We used the PKB/Akt1 kinase inhibitor H-89 as initial reference compound, since we had identified H89 as an effective HDT with antimicrobial activity against *Mtb* and *Stm* previously[1]. Optimization data for fluorescent reporters expressed in *Stm* and *Mtb* is described below. Importantly, our novel medium-throughput flow cytometry-based assay to screen compound and siRNA libraries allows accurate determination of *Mtb* bacterial load within 24h and 72h, respectively, which greatly shortens the time to readout compared to the classical 3-4 week CFU assay for *Mtb*. HeLa (cervical carcinoma) and MelJuSo (melanoma) human cell lines were selected as host models for *Stm* and *Mtb* infection, respectively. In contrast to non-phagocytic HeLa cells, melanocytes were previously reported to possess phagocytic capacity[2], a prerequisite for uptake of mycobacteria. Conversely, MelJuSo was not found to be a suitable target cell line for *Stm* infection as *Stm* did not propagate well in these cells, in line with the previously reported aberrant phenotype of *Stm* in MelJuSo[3]. As chemical compounds may exhibit auto-fluorescence and therefore may cause false positive results when detecting increases in bacterial load, the assay for screening chemical compounds is ideally suited for detecting decreases in bacterial loads.

In HeLa cells infected with genetically-tagged DsRed-*Stm* both DsRed 'bright' and 'dim' infected cell populations were observed (Supplementary Figure 1a). H-89 treatment markedly diminished the DsRed 'bright' population. Since H-89 treatment effectively reduced *Stm* bacterial numbers in HeLa cells as measured by CFU (Supplementary Figure 1e)[1], this DsRed 'bright' population represents cells containing proliferating bacteria. The reduction in *Stm* bacterial load by H-89 treatment was corroborated by fluorescence microscopy (Supplementary Figure 1b). Similarly, *Mtb* infection of MelJuSo cells could be visualized using flow cytometry (Supplementary Figure 1c) and H-89 also decreased the bacterial load in this infection model (Supplementary Figure 1f). Importantly, since alveolar macrophages are the primary target cells for *Mtb in vivo*, we verified that infection of these cells can be similarly visualized using flow cytometry (Supplementary Figure 1d) and that H-89 treatment decreased bacterial loads in primary human pro-inflammatory (Mφ1) as well as anti-inflammatory (Mφ2) cells similar to MelJuSo cells (Supplementary Figure 1g). However, we observed considerable batch-to-batch variation in the proportion of infected macrophages, further supporting the use of the homogenous MelJuSo cell line as an infection model for screening.

We next optimized both the reverse siRNA transfection strategy and bacterial infection conditions by varying cell density, multiplicity of infection (MOI), infection time point and the harvesting time point for analysis using the optimal fluorescent reporters in a medium-throughput setting (outlined below). Using the optimized conditions, knockdown of AKT1 resulted in a significant decrease of both *Stm* and *Mtb* survival in infected cells (Supplementary Figure 1h), but again less so for *Mtb* than for *Stm*, mimicking the effect of treatment of infected cells with AKT1 inhibitor H-89 (Supplementary Figures 1e and f).

In summary, we conclude that HeLa and MelJuSo cells represent new human model systems to study

intracellular *Stm* and *Mtb* infection, respectively, providing novel models for medium-throughput screening of host-directed compounds and genetic manipulation to increase our understanding and treatment of intracellular bacterial infections. Importantly, our novel medium-throughput flow cytometry-based assay allows accurate determination of intracellular *Mtb* bacterial load in compound or siRNA treated cells within a significantly shorter time (2-3 days) window than classical CFU assays (3-4 weeks). The assay is suitable for *Stm, Mtb* and possibly other intracellular bacterial infection models, despite the vast differences in their intracellular 'lifestyles' and replication rates (20 minutes and 18 hours for *Stm* and *Mtb*, respectively)[4-7].

Optimization of fluorescent reporters for flow cytometry-based quantitation of bacterial infection.

To optimize our assay we explored different fluorescent reporters for expression in *Stm* or *Mtb*. Firstly, we monitored the long-term expression kinetics of GFP and DsRed transcribed from plasmids with an identical backbone in *Mtb* (Supplementary Table 1). Despite hygromycin selection, the *Mtb* culture gradually lost GFP expression over time, whereas DsRed expression remained unaltered (Supplementary Figure 2a). As loss of fluorescence would be detrimental to a flow-cytometry-based assay, GFP was excluded as a suitable fluorescent reporter in *Mtb*.

As demonstrated in Supplementary Figure 1, constitutively expressed, stable DsRed constructs provided an excellent assay window to reliably evaluate the effect of chemical compound treatment on bacterial loads of both *Stm*- and *Mtb*-infected cells. Compared to compound treatment experiments, quantification of bacterial infection in siRNA transfected cells often resulted in more subtle phenotypes requiring further assay optimization: while the constitutive expression and high stability of fluorescent reporters can negatively impact the sensitivity of fluorescence-based bacterial growth inhibition assays, this can be overcome by employing conditionally expressed or destabilized fluorescent reporters (decreasing the half-life of DsRed from 4.6 days to several hours)[8,9]. To this end, different fluorescent reporter construct variants (Supplementary Table 1) were expressed in *Stm* (constitutively-expressed stable DsRed, low pH-inducible expressed stable DsRed, or low pH-inducible expressed destabilized DsRed) or in *Mtb* (constitutively-expressed stable DsRed or constitutively-expressed destabilized DsRed) and these bacteria were subsequently used in our HeLa-*Stm* and MelJuSo-*Mtb* infection models following AKT1 silencing. As demonstrated in Supplementary Figure 2b, a low pH-inducible expressed stable DsRed variant increased the assay window in *Stm*-infected HeLa cells following AKT1 silencing (Z' = 0.70) compared to constitutively-expressed stable DsRed (Z' = 0.58) and low pH-inducible expressed destabilized DsRed (Z' = 0.45). The effect of AKT1 silencing in *Mtb*-infected MelJuSo cells could only be visualized using an *Mtb* strain expressing a destabilized DsRed variant (Supplementary Figure 1h), demonstrating that employing this novel fluorescent reporter overcomes a major limitation of fluorescent signal-based growth inhibition assays for slowly replicating bacteria.

HeLa-*Stm* and MelJuSo-*Mtb* infection models combined with a flow cytometry-based readout for intracellular bacterial load allow medium-throughput screening of siRNA libraries.

We next further optimized both the reverse siRNA transfection strategy and bacterial infection conditions by varying cell density, multiplicity of infection (MOI), infection time point and the harvesting time point for analysis using the optimal fluorescent reporters in a medium-throughput setting (96-well format). As shown in Supplementary Figure 3a, knockdown of AKT1 was highly efficient in both HeLa and MelJuSo cells, routinely

resulting in 87-97% knockdown at 72 hours post transfection. To determine an optimal infection window, knockdown kinetics were assessed until 96 hours post transfection. The largest decrease in AKT1 protein levels was observed between 48 and 72 hours post transfection, concurring with the reported 6 to 36 hour half-life of AKT1[10,11] (Supplementary Figure 3b). AKT1 knockdown followed identical kinetics in both HeLa and MelJuSo cells. As cell over-confluence was observed at 96 hours post transfection the assay was not extended beyond this time point. By varying both the time point of infection (24-72 hours post transfection) and the time between infection and readout (24-72 hours) within a 96 hour timeframe, we determined that the optimal assay window for *Stm* infections was obtained when HeLa cells were infected 72 hours post transfection followed by a 24 hour incubation until readout by flow cytometry, while *Mtb* infections resulted in the largest possible assay window when MelJuSo cells were infected 24 hours post transfection followed by a 48 hour incubation until readout by flow cytometry (Supplementary Figure 3b).

Screening assay window, reproducibility, uniformity and validation.

To further confirm assay uniformity and reproducibility, plate uniformity assays were performed and the assay conditions were optimized according to any drift or edge effects that were observed. Results from representative 96-well plates using the optimized screening conditions for *Stm* and *Mtb* are displayed in Supplementary Figure 4a, indicating that the assay generates highly uniform results within the assay plates. The assay yielded an assay window (expressed as a Z' factor) of 0.87 for infections with *Stm* and 0.91 for *Mtb*, greatly exceeding the minimal acceptable Z' factor of 0.4 (Supplementary Figure 4b). In addition, inter-plate reproducibility was high for both infection models (r^2 = 0.82 for *Stm* infections and r^2 = 0.84 for *Mtb* infections) (Supplementary Figure 4c).

Development and use of an *in silico* model for predicting compound activity.

Machine learning in a nutshell.

Machine learning studies computer programs/algorithms that have the ability to learn (improve with experience) where the experience is given in the form of data examples (instances). The input to a typical machine learning algorithm is a single flat table comprising a number of records (rows) and attributes (columns). In general, each row represents an object and columns represent properties of objects[12]. An excerpt of the data table that we used to learn a predictive model is given in Supplementary Table 4. Here, rows correspond to individual compounds and columns contain different properties of these compounds, including bioactivity profiles retrieved from PubChem, intracellular bacterial survival z-scores and host cell viability z-scores. The task formulated here is to predict the intracellular bacterial survival and the host cell viability z-scores for a novel compound using the information from its PubChem bioactivity profile. In machine learning terminology, this translates into a predictive modelling task (or supervised learning) where the two z-scores are called target (or output or dependent) variables/attributes and the variables describing the bioactivity profile are called descriptive (or input or independent) variables/attributes. Furthermore, considering that there are two numeric target variables, the task at hand is called multi-target regression[13]. This is illustrated in the data excerpt in Supplementary Table 4. The output of a data mining algorithm is typically a predictive model (or a set of predictive models) valid for the given data. The dataset used to learn the models is usually called training dataset. The model can then be applied to a different set of data, usually

called testing dataset.

Data pre-processing

In this study, the training set of compounds consisted of our reference compound H-89 and the LOPAC library compounds that were screened in our HeLa-*Stm* and MelJuSo-*Mtb* infection models, while the testing set consisted of all other compounds available in the PubChem public repository. We performed separate analyses on the *Mtb* and *Stm* datasets, but the pre-processing of the data and the data analysis were performed following identical procedures. A schematic overview of the complete pre-processing pipeline is displayed in Supplementary Figure 5a.

The first step of the data pre-processing was to uniquely identify the LOPAC compounds by linking them to their corresponding PubChem IDs. Based on the structure-data format (SDF) information provided by the compound supplier, we linked the LOPAC compounds to compounds from PubChem. To this end, SDF information of the compounds was first converted into InChIKey using the OpenBabel toolbox (http://www.openbabel.org) and then mapped to PubChem IDs. Next, we manually checked whether the mapping was correct and provided manual mapping where the InChIKey information was not sufficient, obtaining a list of PubChem compounds that were used in our study. Next, biological activity information was retrieved for the LOPAC compounds from each compounds' 'bioassays' section in PubChem. From the bioassays, only human protein targets for which compounds were confirmed to be active were extracted, yielding a total of 1058 protein targets. This resulted in the columns on the left-hand side of Supplementary Table 4 (the descriptive variables). At the end of the pre-processing pipeline, each compound is described with both its protein targets (as descriptive attributes for machine learning) and experimental measurements of activity and viability (as target attributes for the machine learning). These compound descriptions comprise our training set. Finally, we considered all of the remaining compounds from PubChem as potential candidates for drug repurposing (Supplementary Figure 5a). We applied the pre-processing pipeline on each of these compounds as described above. Only compounds confirmed to target at least one of the 1058 human target proteins were included, thus obtaining a testing set of 460,580 compounds. Note that the compounds from the testing set have information only for the bioactivity profiles (the descriptive attributes), while the intracellular bacterial survival and host cell viability z-scores are not known but the goal is to predict these. We obtained these predictions by applying the predictive model (predictive clustering tree) learned from the training data to each of the compounds from the testing set, as described in more detail below.

Predictive clustering trees

To analyze the data and learn a predictive model, we used the machine learning tool CLUS (available at http://clus.sourceforge.net). Specifically, we used predictive clustering trees (PCTs) for multi-target regression as models[13,14]. PCTs are a generalization of regression trees, a machine learning approach commonly used for regression. An example PCT is shown in Supplementary Figure 5c. Similar to regression trees, PCTs are tree-like structures that have internal nodes and leaves. The internal nodes contain tests on the descriptive variables (i.e. asking whether a given protein is targeted or not), while leaves give predictions for the target variables (the predicted z-scores for intracellular bacterial survival and host cell viability). We opted to use PCTs because they are able to implicitly exploit the relation between the target variables during model construction.

Furthermore, PCTs are easily interpretable. A PCT can be viewed as a hierarchy of clusters with each node corresponding to a cluster. The top-node of a PCT corresponds to one cluster (group) containing all data points. This cluster is recursively partitioned into smaller clusters while moving down the tree. The leaves represent the clusters at the lowest level of the hierarchy and each leaf is labeled with its cluster's centroid/prototype (the averages of the target variables are the prediction made by the leaf).

PCTs are built with a greedy recursive top-down induction algorithm. This learning algorithm starts by selecting a test for the root node by using a heuristic function computed on the training examples. The goal of the heuristic is to guide the algorithm towards small trees with good predictive performance. Based on the selected test, the training set is partitioned into subsets according to the test outcome. This is recursively repeated to construct the subtrees. The partitioning process stops when a stopping criterion is satisfied (i.e. the minimal number of examples per leaf is reached or the heuristic score no longer changes). In that case, the prototype (the prediction) is calculated as the averages of the target variables and stored in a leaf.

Ensembles of PCTs

An ensemble is a set of predictive models (called base models). The prediction of an ensemble for a new example is obtained by combining the predictions of all base models from the ensemble. These predictions can be combined by averaging them. The ensemble learning procedure is illustrated in Supplementary Figure 5d. Here, we consider ensembles of PCTs for multi-target regression[13]. For constructing the base models, we used the Bagging method[15]. Bagging is an ensemble method that constructs the base models in the ensemble by making bootstrap samples (E_i) of the training set (also called bootstrap replicates) and using each of these replicates to construct a predictive model. Each bootstrap sample is obtained by randomly sampling training instances, with replacement, from the original training set, until an equal number of instances as in the training set is obtained.

Reliability scores

A very important aspect of using a predictive model is the ability to estimate the reliability of the predictions it makes. This reliability indicates how confident the model is about its prediction. Ensembles offer a natural way of estimating the reliability of their predictions by exploiting their voting mechanism[16]. When a prediction is made for an unlabeled example (these are examples that do not have z-score values for intracellular bacterial survival and host cell viability) by an ensemble, we consider it reliable if the predictions of the individual models in the ensemble are coherent, i.e., if the variance of the predictions is low. Here, we get the reliability score for a prediction of two targets by averaging the variances of the predictions for each of the two targets (the variances of the predicted z-scores for intracellular bacterial survival and host cell viability).

Data analysis workflow

To identify candidate compounds in the set of testing compounds to screen in our MelJuSo-*Mtb* or HeLa-*Stm* infection models, we followed the data analysis workflow outlined in Supplementary Figure 5b. First, we used the training dataset to construct a predictive model (a PCT) using a data-mining algorithm (the PCT algorithm). Next, the predictive model was applied to the testing set to obtain the predictions for the activity of the compounds, expressed as z-scores. Finally, we calculated a reliability score for each prediction for a test compound.

This data analysis workflow resulted in a small set of selected candidate compounds from all of the 460,580 compounds in the testing set. Predicted *Mtb* hits were defined as compounds with a predicted intracellular bacterial survival z-score below -2 and a host cell viability z-score between -1 and 1 with a prediction reliability greater than 0.5, or an intracellular bacterial survival z-score below -1.75, a host cell viability z-score between -0.75 and 0.75 and a prediction reliability higher than 0.75. This yielded a total of 47 candidate compounds (Supplementary Table 5). Predicted *Stm* hits were defined as compounds with a predicted intracellular bacterial survival z-score below -2, a host cell viability z-score between -1 and 1 and a prediction reliability greater than 0.5 or an intracellular bacterial survival z-score below -1.5, a host cell viability z-score between -0.75 and 0.75 and a prediction reliability higher than 0.5. This yielded a total of 30 candidate compounds (Supplementary Table 6). From the resulting lists of predicted hits, compounds were then selected for further experiments based on their commercial availability.

Generation of a mycobacterial destabilized DsRed construct and expression in *Mtb* H37Rv

The destabilized DsRed gene (DsRed C-terminally fused to amino acids 422-461 of the mouse ornithine decarboxylase (MODC) to induce ubiquitin-independent proteasomal degradation[9]) was amplified from the pMW266[PpagC/destabilized DsRed] plasmid by PCR and cloned into the Gateway (Invitrogen) adapted mycobacterial expression plasmid pSMT3[17]. In this vector, expression of destabilized DsRed is constitutive and controlled by the hsp60 promoter. Electrocompetent *Mtb* H37Rv were freshly prepared from a 100 ml log-phase culture by incubation at 4°C for 90 minutes followed by suspension of the bacteria in 1 ml ice cold PBS containing 10% glycerol. 100 µl Bacterial suspension was then transformed with 1 µg plasmid DNA by electroporation. Transformed bacteria were suspended in 10 ml 7H9 broth, incubated overnight at 37°C and subsequently plated on Difco Middlebrook 7H10 agar (Becton Dickinson, Breda, The Netherlands) under Hygromycin (50 µg/ml) selection (Life Technologies-Invitrogen, Bleiswijk, The Netherlands). DsRed expression of individual clones was verified by flow cytometry.

Screening assay validation and screening statistics

The flow cytometry-based screening assay for *Stm* and *Mtb* infection of human cell lines was developed adhering to guidelines published by the NIH Chemical Genomics Center[18]. Cells were transfected and infected with *Stm* or *Mtb* in flat-bottom 96-wells plates as described in the Experimental Procedures of the main manuscript. Cells were harvested by trypsinization and fixed with 1% paraformaldehyde prior to readout using a FACSCalibur (Becton Dickinson) with high-throughput sampler (HTS) extension (Becton Dickinson). Data was analyzed using FlowJo for Mac OS X version 8.8.7 (TreeStar, Ashland, OR, USA) and both the total and bright DsRed positive populations expressed as a frequency of the parent forward/side-scatter gate and the total event counts were extracted for further analysis. Z' factors (to determine the assay window) were calculated using the formula $Z' = \frac{\left(AVG_{\text{DMSO}} - \frac{3SD_{\text{DMSO}}}{\sqrt{n}}\right) - \left(AVG_{\text{H-89}} + \frac{3SD_{\text{H-89}}}{\sqrt{n}}\right)}{AVG_{\text{DMSO}} - AVG_{\text{H-89}}}$, where AVG is the average percentage of DsRed positive events measured after DMSO or H-89 treatment, SD is the standard deviation of these measurements and n is the number of replicates. Z-scores were calculated using the formula $z = \frac{x - AVG_{DMSO}}{STDEV_{\text{DMSO}}}$, where the difference between the percentage of DsRed positive events (bacterial load) or the total event count (cell viability) of a single replicate of an experimental condition (x) and the average percentage of DsRed positive

events or the total event count of the DMSO control (AVG_{DMSO}) is divided by the standard deviation of the DMSO control ($STDEV_{DMSO}$). An average z-score ≤ -2 or ≥ 2 was used as a hit cut-off, unless otherwise indicated.

Immunoblotting

Cells were lysed by heating in loading buffer (250 mM Tris, 8% w/v SDS, 20% glycerol, 20% β-mercaptoethanol and 0.002% w/v bromophenolblue) for 5 minutes at 99°C. Proteins from lysates of 50,000 cells were mass-separated by SDS-PAGE gel electrophoresis and subsequently blotted on a PVDF membrane. Following fixation in pure methanol for 15 seconds at RT, membranes were blocked for 1 hour at RT with 5% w/v milk. Blots were then incubated overnight at 4°C with mouse anti-human AKT1 IgG1 (1:5,000; Cell Signaling Technology, Leiden, The Netherlands) diluted in 5% w/v milk. After incubation, membranes were washed for 1 hour at RT with PBS containing 0.1% Tween-20, refreshing the wash buffer every 10 minutes. Blots were incubated with HRP-labelled goat anti-mouse IgG (1:12,500; Thermo Scientific, Bleiswijk, The Netherlands) and HRP-labelled goat anti-human actin (1:80,000; Santa Cruz, Heidelberg, Germany) diluted in 5% w/v milk for 1 hour at RT and washed as above. Protein bands were visualized on a photosensitive film by Enhanced ChemiLuminescence (ECL Plus, Amersham-GE Healthcare, Freiburg, Germany). Relative protein abundance was quantified by calculating the area under the curve (AUC) for each band using ImageJ (version 1.43n) and each lane was normalized against the AUC of the actin band.

Compound identification within the PubChem repository and retrieval of BioAssay data

Structure-data format (SDF) data supplied with the LOPAC library was converted to InChIKey using the OpenBabel toolbox (http://www.openbabel.org). InChIKeys were subsequently mapped to PubChem IDs and correct identification was checked manually. Compounds were manually linked to PubChem IDs if InChIKey information was insufficient for automated identification. For each of the identified compounds, BioAssay data was retrieved from the PubChem repository (as of July 25th, 2014). Human protein targets for which compounds were confirmed to be active were then extracted from the BioAssay data. Compounds were subsequently described with their confirmed protein targets, as well as z-scores for bacterial load and cell viability from the primary screening data. All remaining compounds in the PubChem repository that were not included in the LOPAC library were described with their confirmed protein targets as above. Compounds that were not confirmed to target any of the protein targets identified for the LOPAC compounds were excluded from analysis and the remaining compounds were used as a testing set for the predictive model.

Predictive model

Using LOPAC compounds as a training set, BioAssay data obtained from PubChem (descriptive variables) were related to the z-scores for bacterial load and cell viability from the primary screening data (target variables) using the predictive modelling approach of multi-target regression to simultaneously predict both target variables. Predictive models were constructed within the predictive clustering framework[14], using predictive clustering trees (PCTs) as predictive models for multi-target regression. Ensembles of predictive clustering trees were generated[13] using the Bagging ensemble learning method[15] as implemented in the data mining tool CLUS (http://clus.sourceforge.net). Multiple predictive models were constructed using different bootstrap samples of the training dataset and their predictions were averaged to obtain an overall prediction. The variance of the predictions for the two target variables across the models in the ensemble was calculated for

each target variable separately, averaged between the two targets and then used as a reliability estimation score[16].

Supplementary Figures

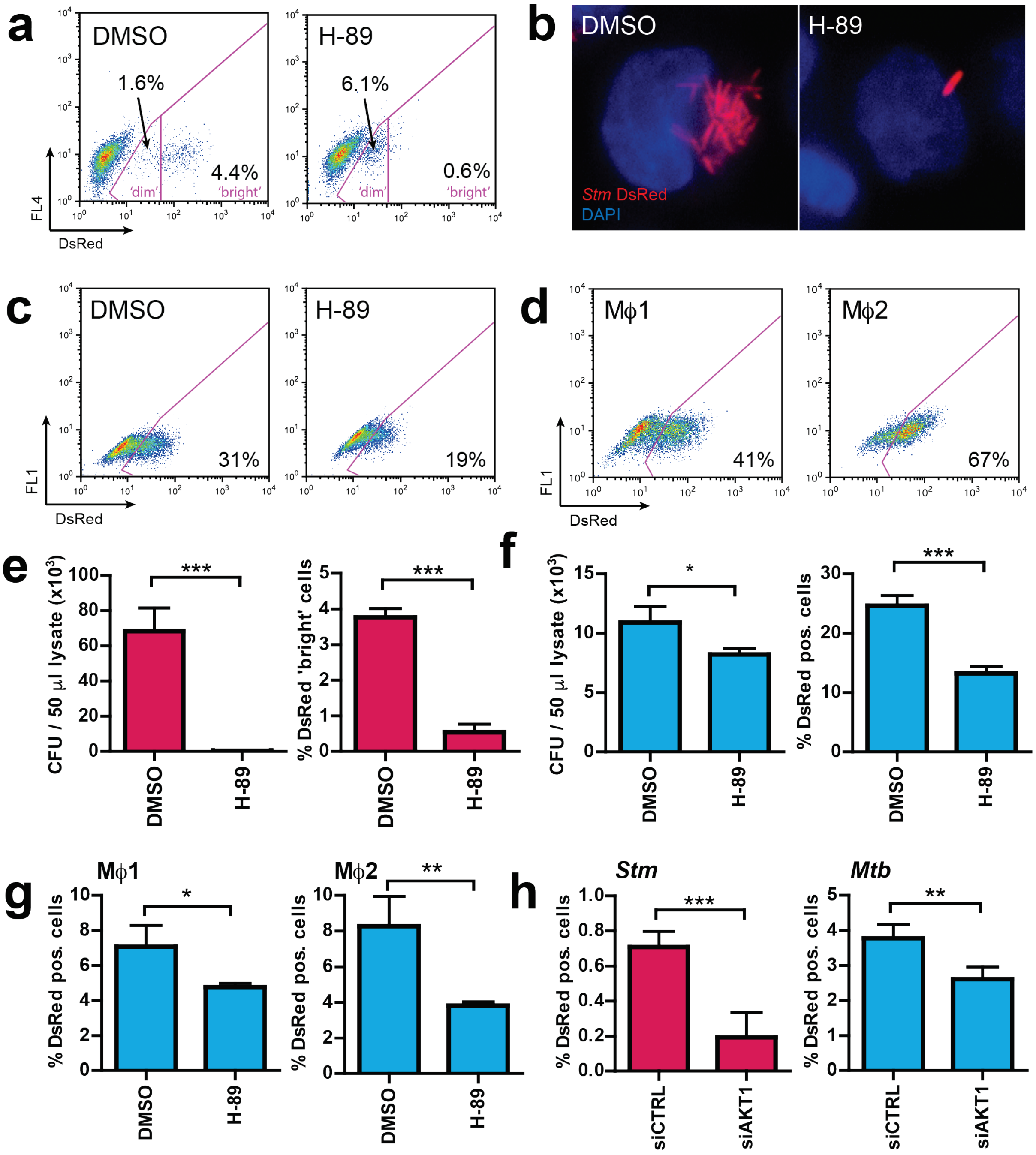

Supplementary Figure 1. A flow cytometry-based readout for intracellular bacterial load.

(a) Flow cytometry gating strategy. Shown are dot plots of HeLa cells infected with *Stm* constitutively expressing stable DsRed and treated with H-89 or DMSO at 10 µM as a negative control. Gates were drawn for separate analysis of total DsRed positive and DsRed 'bright' populations. Percentages of DsRed positive events in each gate are indicated. (a) Fluorescence microscopy of HeLa cells infected and treated as in (a). A representative infected cell is shown for both conditions. (c) Flow cytometry gating strategy for MelJuSo cells infected with *Mtb* constitutively expressing stable DsRed and treated as in (A). Percentages of DsRed positive events are indicated. (d) Flow cytometry of human primary Mφ1 (left panel) and Mφ2 (right panel) macrophages infected with *Mtb* constitutively expressing stable DsRed. Percentages of DsRed positive events are indicated. (e) Comparison of CFU assay (left panel) to the flow cytometry-based screening assay (right

panel) for HeLa cells infected and treated as in (a). A representative result of 3 experiments is shown. Bars display mean ± standard deviation. Statistical significance was tested using a t-test. (f) Comparison of CFU assays (left panel) to the flow cytometry-based screening assay (right panel) is shown for MelJuSo cells infected and treated as in (c). A representative result of 3 experiments is shown. Bars display mean ± standard deviation. Statistical significance was tested using a t-test. (g) Flow cytometry of Mφ1 and Mφ2 cells infected and treated as in (c). Bars display mean ± standard deviation. Statistical significance was tested using a t-test. (h) Infection of AKT1-silenced HeLa or MelJuSo cells with *Stm* expressing low pH-inducible, stable DsRed (left panel) and *Mtb* constitutively expressing destabilized DsRed (right panel), respectively, analyzed by flow cytometry. Bars display mean ± standard deviation. Statistical significance was tested using a t-test. Shown are results of 6 replicate samples from 1 representative screening plate out of more than 20 replicate plates.

(* = p-value <0.05, ** = p-value < 0.01, *** = p-value < 0.001).

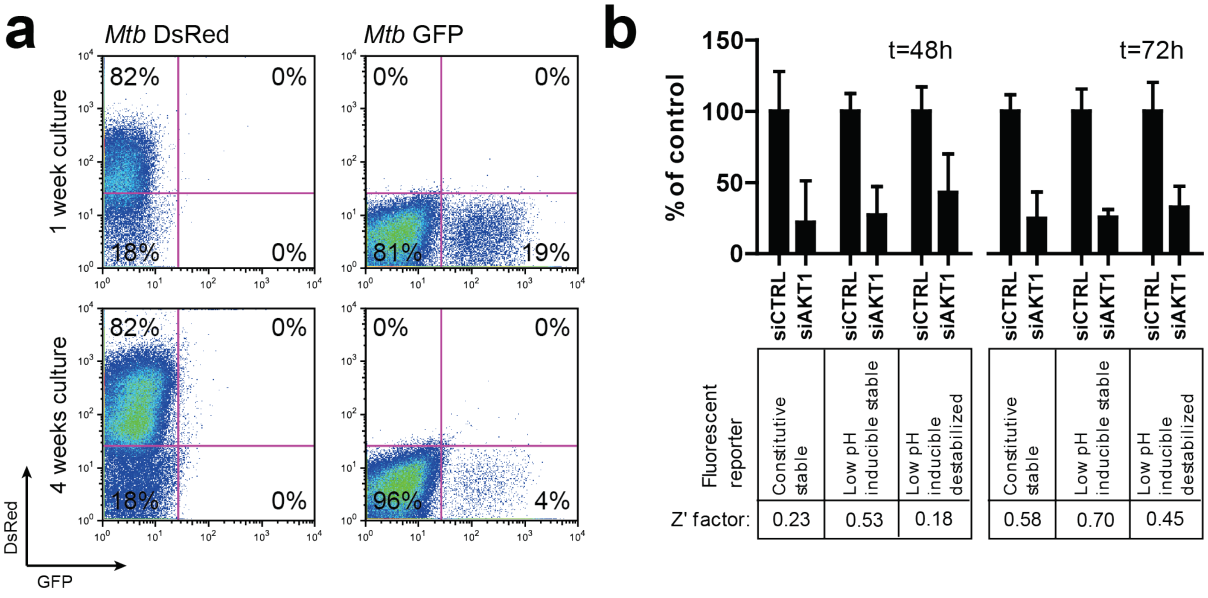

Supplementary Figure 2. Optimization of the flow cytometry-based assay to monitor bacterial load using different fluorescent reporters.

(a) Flow cytometric analysis of DsRed (left panel) and GFP (right panel) expression in *Mtb* cultured for either 1 week (top panel) or 4 weeks (bottom panel) after thawing of a frozen batch. (b) Infection of HeLa cells using *Stm* strains expressing different fluorescent reporters (constitutive stable, low pH-inducible stable and low pH-inducible destabilized DsRed) at 48 hours (left panel) or 72 hours (right panel) post transfection with the indicated siRNA oligos. siCTRL: scrambled siRNA. The upper panel gives the level of inhibition of intracellular *Stm* in siAKT1 silenced cells expressed as a percentage of the control (siCTRL treated) condition ± standard deviation. The signal window resulting from infection with the indicated fluorescent *Stm* strains is expressed as a Z' factor.

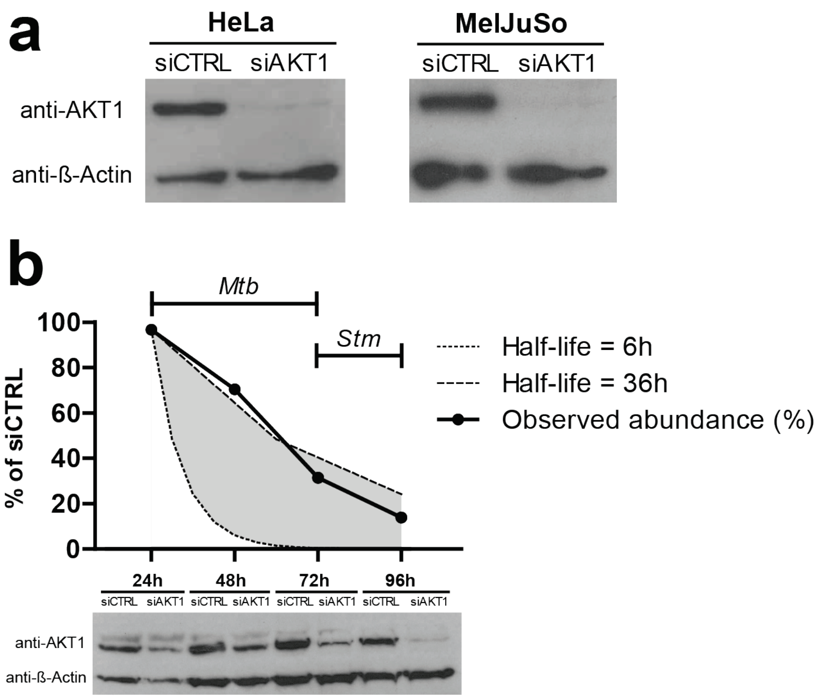

Supplementary Figure 3. Knockdown of AKT1 in HeLa and MelJuSo cells.

(a) Western blot showing AKT1 knockdown (siAKT1) compared to scrambled siRNA (siCTRL) in HeLa (left panel) and MelJuSo (right panel) whole cell lysates at 72 hours post transfection. β-Actin was included as loading control. (b) Time course of AKT1 silencing by western blot analysis, normalized for β-Actin. AKT1 protein abundance is shown relative to cells transfected with scrambled siRNA between 24 to 96 hours post transfection (black line). The dotted and dashed lines represent theoretical 6 to 36-hour half-lives reported for AKT1, respectively. The horizontal bars depict the infection time windows for both *Stm* and *Mtb* used in the final screening assay.

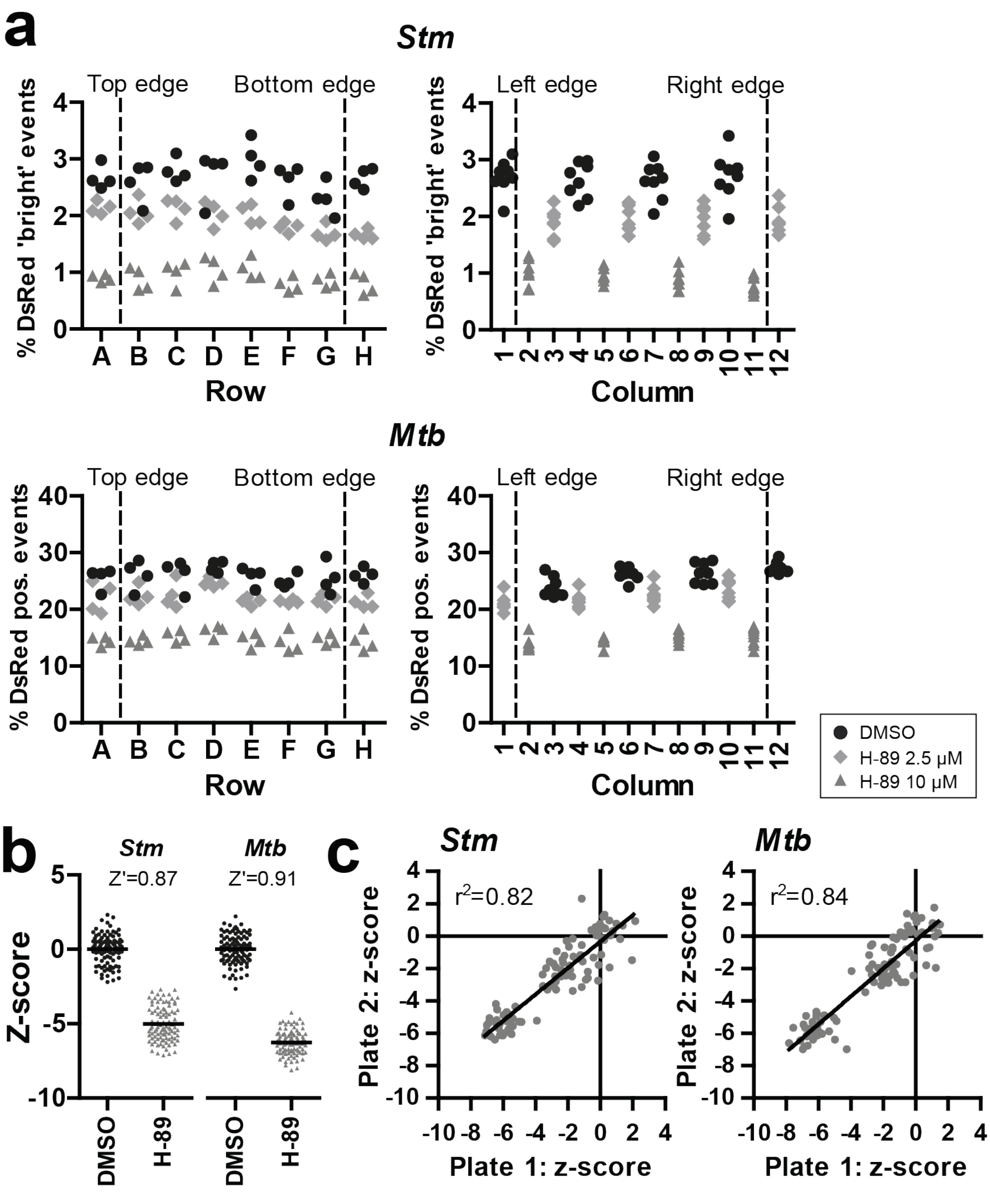

Supplementary Figure 4. Screening assay window, reproducibility, uniformity and validation.

(a) Plate uniformity test using HeLa cells infected with *Stm* constitutively expressing stable DsRed (top panel) or MelJuSo cells infected with *Mtb* constitutively expressing stable DsRed (bottom panel) and treated with 2.5 µM H-89, 10 µM H-89 or DMSO at equal v/v. The percentage of gated 'bright' (top panel) or total (bottom panel) DsRed positive events from individual wells were grouped either by row (left panel) or by column (right panel). The dashed lines indicate the wells on the edges of the plates to identify edge effects. (b) Assay windows for both the HeLa-*Stm* and the MelJuSo-*Mtb* infection models (as in A) following assay optimization. Z' factors are displayed for each infection model. Shown are 96 individual replicates of infected cells treated with 10 µM H-89 or DMSO. Percentages of DsRed 'bright' cells (HeLa-*Stm*) and DsRed positive cells (MelJuSo-*Mtb*) are expressed as a z-score. (c) Comparison of individual plates from plate uniformity tests for HeLa-*Stm* (left panel) and MelJuSo-*Mtb* (right panel) infection models (as in A). Z-scores were plotted for individual wells and a correlation coefficient was calculated by linear regression.

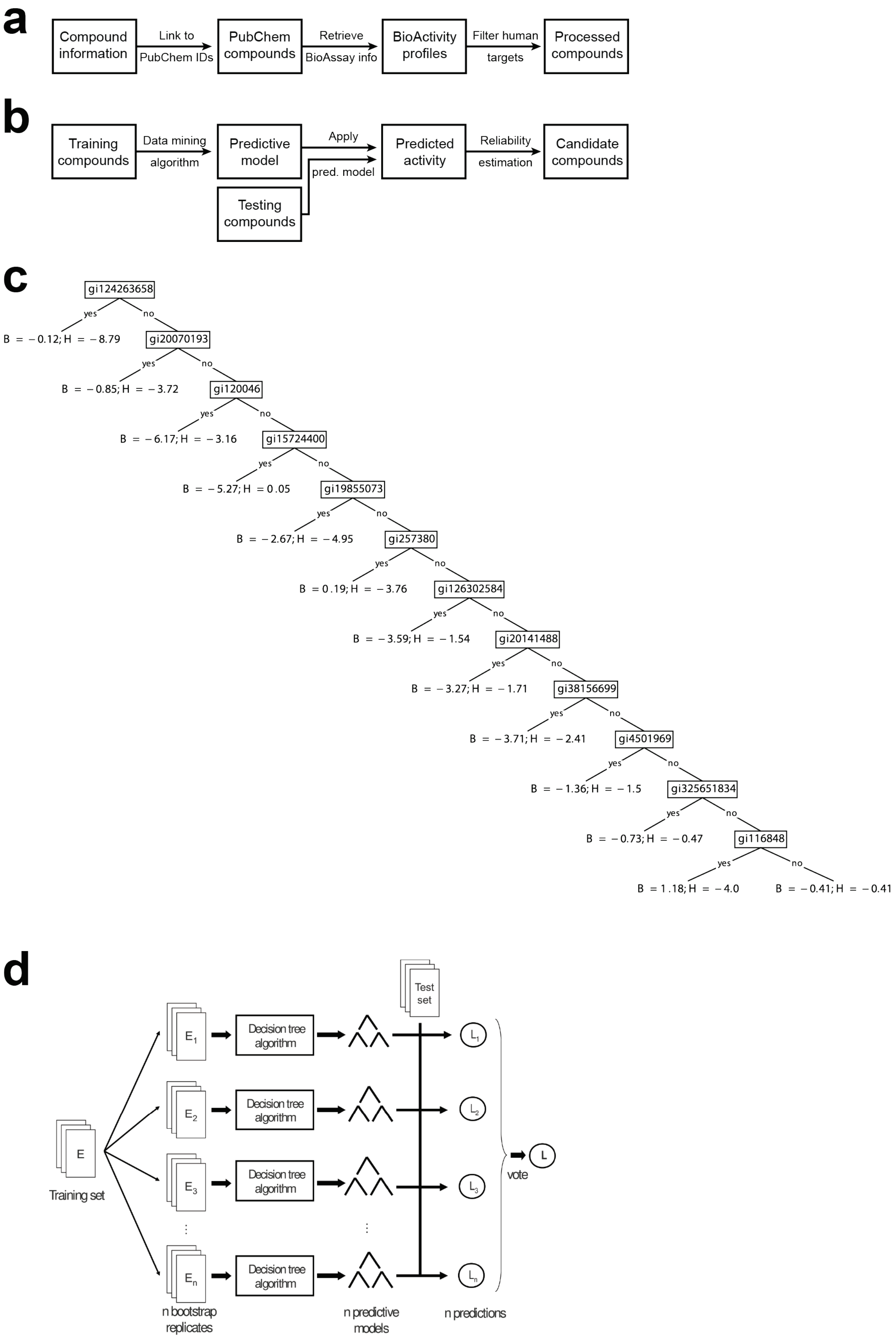

Supplementary Figure 5. Data pre-processing pipeline and predictive model methods.

(a) Pre-processing pipeline used to link compounds described by structured-data files to compounds in the

PubChem database of compounds. (b) Data analysis pipeline from the pre-processed compounds to the new candidate compounds for wet-lab experiments. (c) Example predictive clustering tree (PCT) obtained from the screening data for *Mtb*. The internal nodes of the tree refer to the descriptive variables and check whether or not a compound targets a given protein. The leaves then give the predictions for the intracellular bacterial survival and the host cell viability z-scores. For example, compounds that target *gi15724400*, but not *gi14263638*, *gi20070193* or *gi120046*, are predicted to drastically reduce bacterial load (z-score of -5.27) and not affect cell viability (z-score of 0.05). (d) An illustration of the ensemble learning method of bagging. From the training set of examples (E), n bootstrap samples are created (E1, E2, ..., En). Predictive models are then constructed (using a tree construction algorithm) on each of the n replicates. The predictions of the base predictive models (L1, L2... Ln) are combined by a voting (averaging) scheme into the final prediction (L) of the ensemble.

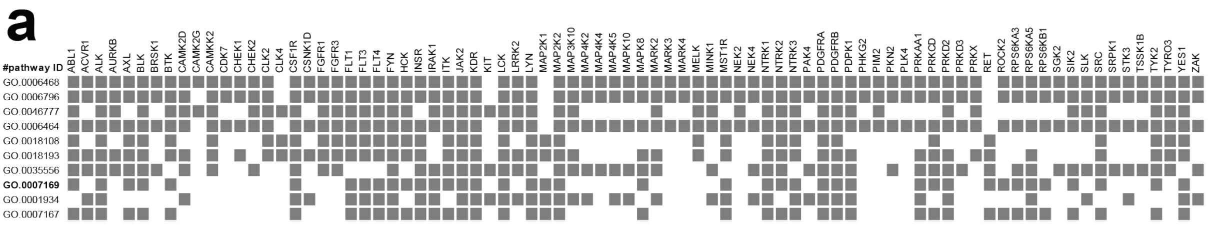

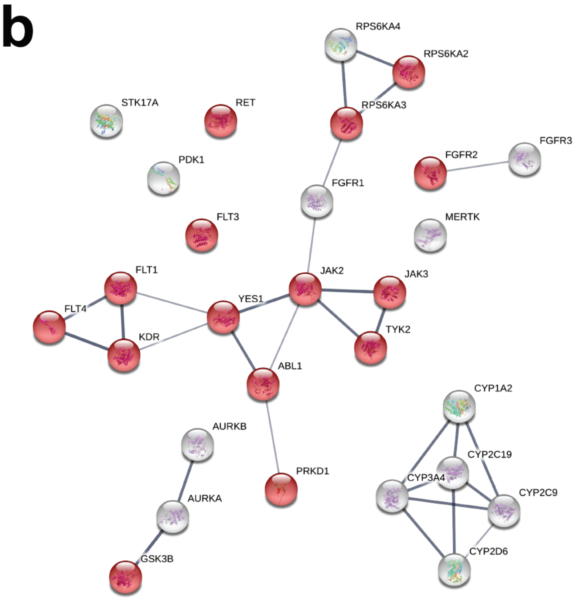

#pathway ID	pathway description	gene count	FDR
GO.0046777	protein autophosphorylation	16	4.31E-22
GO.0006468	protein phosphorylation	21	2.83E-21
GO.0018108	peptidyl-tyrosine phosphorylation	14	1.18E-19
GO.0018193	peptidyl-amino acid modification	20	3.37E-19
**GO.0007169**	**transmembrane receptor protein tyrosine kinase signaling path**	**15**	**1.11E-12**
GO.0016098	monoterpenoid metabolic process	5	2.27E-11
GO.0010941	regulation of cell death	16	1.44E-09
GO.0035556	intracellular signal transduction	17	2.08E-09
GO.0070887	cellular response to chemical stimulus	18	4.47E-09
GO.0038084	vascular endothelial growth factor signaling pathway	5	4.75E-09

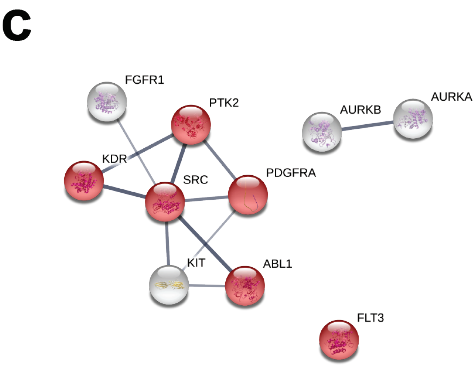

#pathway ID	pathway description	gene count	FDR
GO.0046777	protein autophosphorylation	10	4.17E-17
GO.0014068	positive regulation of phosphatidylinositol 3-kinase signaling	6	3.24E-10
GO.0018108	peptidyl-tyrosine phosphorylation	7	4.63E-10
GO.0006468	protein phosphorylation	9	2.33E-09
GO.0018193	peptidyl-amino acid modification	9	3.81E-09
GO.0007173	epidermal growth factor receptor signaling pathway	6	2.41E-07
GO.0038093	Fc receptor signaling pathway	6	3.67E-07
GO.0043552	positive regulation of phosphatidylinositol 3-kinase activity	4	4.92E-07
GO.0038083	peptidyl-tyrosine autophosphorylation	4	1.80E-06
GO.0043067	regulation of programmed cell death	8	5.99E-06
GO.0043410	positive regulation of MAPK cascade	6	6.54E-06
GO.0043069	negative regulation of programmed cell death	7	7.76E-06
GO.1900274	regulation of phospholipase C activity	4	1.30E-05
GO.0038084	vascular endothelial growth factor signaling pathway	3	1.56E-05
GO.0035556	intracellular signal transduction	8	3.30E-05
GO.0006935	chemotaxis	6	4.12E-05
GO.2000145	regulation of cell motility	6	4.47E-05
**GO.0007169**	**transmembrane receptor protein tyrosine kinase signaling pathway**	**6**	**6.47E-05**

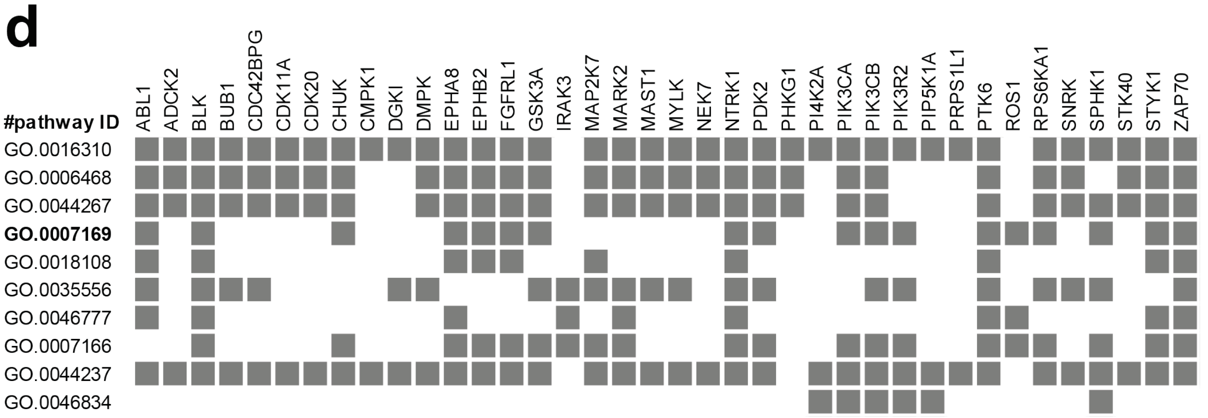

Supplementary Figure 6. STRING analysis of targets of Dovitinib, AT9283, ENMD-2076 and siRNA screening hits.

(a) Association of individual targets of Dovitinib with the top 10 enriched GO terms is indicated by filled squares. (b) STRING network of potential targets of AT9283 retrieved from the ChEMBL repository Target Summary section (top panel). Individual proteins are displayed as nodes. Lines represent protein-protein interactions and the thickness of the lines indicates confidence. Proteins participating in the 'transmembrane receptor tyrosine kinase signaling pathway' are displayed in red. The top 10 enriched GO terms in the 'Biological Function' category are displayed along with the number of genes/proteins annotated with the

indicated GO terms and the false discovery rate (FDR) of the enrichment (bottom panel). (c) STRING network of potential targets of ENMD-2076 retrieved from the ChEMBL repository Target Summary section (top panel) and the top 18 enriched GO terms in the 'Biological Function' category (bottom panel) are displayed as in (b). (d) Association of individual siRNA hit kinases with the top 10 enriched GO terms is indicated by filled squares.

Supplementary Tables

Supplementary Table 1. Bacterial strains, plasmids used for fluorescent protein expression and their respective antibiotic selection markers.

Base strain	*Plasmid*	*Selection (concentration)*
Salmonella enterica serovar Typhimurium SL1344.	pMW211[C.10E/DsRed] (Constitutive promoter).	Ampicillin (100 µg/ml).
Salmonella enterica serovar Typhimurium SL1344.	pMW215[PpagC/DsRed] (Low-pH inducible promoter).	Ampicillin (100 µg/ml).
Salmonella enterica serovar Typhimurium SL1344.	pMW266[PpagC/destabilized DsRed] (Low-pH inducible promoter).	Ampicillin (100 µg/ml).
M. tuberculosis H37Rv.	pSMT3[Phsp60/DsRed].	Hygromycin (50 µg/ml).
M. tuberculosis H37Rv.	pSMT3[Phsp60/GFP].	Hygromycin (50 µg/ml).
M. tuberculosis H37Rv.	pSMT3[Phsp60/destabilized DsRed].	Hygromycin (50 µg/ml).
MDR M. tuberculosis Beijing family China 16319 (Kremer 43)		
MDR M. tuberculosis Dutch outbreak 2003-1128		

Supplementary Table 2. LOPAC MelJuSo-*Mtb* primary screen hits using a bacterial load cut-off at z<-2 and a host cell viability cut-off at z>-2.

Bacterial load z-score	Cell viability z-score	Compound name
**-6.02**	**-1.36**	**SB 216763**
**-5.79**	**-0.33**	**SU 6656**
**-5.25**	**-1.14**	**Quinacrine dihydrochloride**
**-4.86**	**0.38**	**GW5074**
-3.87	-0.14	3',4'-Dichlorobenzamil hydrochloride
**-3.83**	**-1.01**	**Tyrphostin AG 494**
-3.77	-1.20	Haloperidol
-3.43	-1.55	Metaproterenol hemisulfate
-3.16	2.35	Serotonin hydrochloride
-3.14	0.96	Hydrocortisone 21-hemisuccinate sodium salt
-2.98	-1.07	Nortriptyline hydrochloride
-2.95	-0.88	LY-294,002 hydrochloride
-2.94	-0.14	Emodin
-2.93	-0.77	NNC 55-0396
-2.92	-0.64	Metrifudil
-2.91	0.76	LY-367,265
-2.78	-1.31	Fluspirilene
-2.75	0.06	nor-Binaltorphimine dihydrochloride
-2.66	-0.38	R-(-)-Fluoxetine hydrochloride
-2.65	-1.30	Loperamide hydrochloride
-2.63	0.08	BU224 hydrochloride
-2.62	-1.23	Nylidrin hydrochloride
-2.57	1.52	Farnesylthiosalicylic acid
-2.50	-0.46	PD 168,077 maleate
-2.48	-1.80	GR 127935 hydrochloride hydrate
-2.48	-0.49	5-Hydroxyindolacetic acid
-2.47	-0.46	Fenoldopam monohydrobromide
-2.47	0.01	S-Nitrosoglutathione
-2.44	-0.04	L-Histidine hydrochloride
-2.43	0.65	L-165,041
-2.41	0.04	4-Hydroxybenzhydrazide
-2.41	-1.83	Forskolin
-2.40	-0.38	AMN082
-2.36	-0.45	NAN-190 hydrobromide
-2.36	-0.68	Labetalol hydrochloride
-2.35	-0.41	Hexahydro-sila-difenidol hydrochloride, p-fluoro analog
-2.35	-0.01	L-Canavanine
-2.33	0.05	BRL 50481
-2.31	-0.11	N-Methyl-beta-carboline-3-carboxamide
-2.31	0.23	B-HT 933 dihydrochloride
-2.30	-1.05	Tyrphostin AG 527
-2.30	-0.52	1,3-Dimethyl-8-phenylxanthine
-2.30	0.10	Dopamine hydrochloride
-2.29	-1.17	A-77636 hydrochloride
-2.28	-1.53	Formoterol fumarate dihydrate
-2.25	-0.22	cis-(Z)-Flupenthixol dihydrochloride
-2.25	0.18	5-hydroxydecanoic acid sodium salt
-2.24	0.64	Isoguvacine hydrochloride
-2.23	-0.38	Nimesulide
-2.23	-0.66	alpha-Lobeline hydrochloride
-2.20	-0.67	Hydroxyurea
-2.19	-1.31	Fenoterol hydrobromide
-2.17	-0.34	L-733,060 hydrochloride
-2.17	-0.05	Minocycline hydrochloride
-2.17	-0.71	3-Nitropropionic acid
-2.17	0.41	LFM-A13
-2.16	-1.01	Nalidixic acid sodium salt
-2.16	-1.07	1,3,5-tris(4-hydroxyphenyl)-4-propyl-1H-pyrazole
-2.16	0.57	CR 2249
-2.15	1.02	p-MPPF dihydrochloride
-2.15	-0.04	Naltrexone hydrochloride
-2.15	0.87	Fluphenazine dihydrochloride
-2.14	-1.08	(-)-Tetramisole hydrochloride
-2.14	-0.31	Hydralazine hydrochloride
-2.14	0.09	(+)-Hydrastine
-2.12	0.54	MHPG sulfate potassium
-2.12	-0.67	6-Hydroxy-DL-DOPA
-2.12	-0.52	Ro 90-7501
-2.12	0.75	Neostigmine bromide
-2.11	-0.20	4-Amino-1,8-naphthalimide
-2.10	0.26	Flunarizine dihydrochloride
-2.09	-0.39	2-Methyl-5-hydroxytryptamine maleate
-2.09	0.01	L-368,899 hydrochloride
-2.09	-0.48	Tyrphostin AG 528
-2.09	-0.50	Lamotrigine
-2.09	-0.38	VER-3323 hemifumarate salt
-2.09	0.51	BU99006
-2.09	0.11	GYKI 52466 hydrochloride
-2.09	0.51	Hexamethonium bromide
-2.09	0.49	Flutamide
-2.07	0.05	Hypotaurine
-2.06	-0.06	NCS-356 sodium salt hydrate
-2.06	-0.07	(±)-7-Hydroxy-DPAT hydrobromide
-2.06	0.88	Hydroxylamine hydrochloride
-2.05	0.14	MDL 26,630 trihydrochloride
-2.04	-0.23	4-Hydroxy-3-methoxyphenylacetic acid
-2.03	-0.35	Fenofibrate
-2.03	-0.02	(±)-8-Hydroxy-DPAT hydrobromide
-2.02	-0.25	5-Hydroxy-L-tryptophan
-2.01	0.78	Methiothepin mesylate

Hit compounds that performed better than H-89 in both the primary screen and the rescreen are displayed in bold.

Supplementary Table 3. LOPAC HeLa-*Stm* primary screen hits using a bacterial load cut-off at z<-2 and a host cell viability cut-off at z>-2.

Bacterial load z-score	Cell viability z-score	Compound name
-4.06	-1.54	Trimethoprim
-3.90	0.98	Haloperidol
**-3.64**	**1.57**	**Mibefradil dihydrochloride**
-3.45	1.21	Ofloxacin
-2.86	1.96	Demeclocycline hydrochloride
-2.70	-0.38	Doxazosin mesylate
-2.47	2.29	Metergoline
-2.30	1.31	Fluspirilene
-2.20	0.52	8-(3-Chlorostyryl)caffeine
-2.00	2.15	GW2974

Hit compounds that performed better than H-89 in both the primary screen and the rescreen are displayed in bold.

Supplementary Table 4. Excerpt from the data table for the *Mtb* screen used to learn the predictive models.

	Descriptive/Input space (PubChem BioAssay accession)					Target/Output space	
PubChem ID	gi:10864009	gi:10880131	gi:10937869	gi:112938	...	Bacterial load z-score	Cell viability z-score
ID1	1[a]	0	0	1		-2.61	0.29
ID2	0	0	0	1		-1.57	-0.43
ID3	0	0	0	0		0.47	0.22
ID4	0	0	1	0		-0.83	-0.13
ID5	1	1	0	0		-2.58	-0.53
ID6	0	1	0	0		1.78	0.97
...	...					...	

[a] '1' indicates that the compound has the corresponding protein as a confirmed target in a PubChem BioAssay.

Supplementary Table 5. Complete list of compounds identified as potential hits from the *Mtb* predictive model output.

PubChem ID	Predicted bacterial load z-score	Predicted cell viability z-score	Reliability
6604502	-2.59	-0.59	0.61
46233889	-2.38	-0.84	0.54
46235770	-2.38	-0.84	0.54
56945171	-2.38	-0.84	0.54
56945172	-2.38	-0.84	0.54
56945173	-2.38	-0.84	0.54
56945174	-2.38	-0.84	0.54
56945175	-2.38	-0.84	0.54
56945277	-2.38	-0.84	0.54
24995659	-2.35	-0.96	0.61
**10113978**	-2.29	-0.91	0.53
**11496629**	-2.27	-0.95	0.54
**10907042**	-2.24	-0.87	0.54
59627005	-2.21	-0.93	0.54
**16041424**	-2.15	-0.89	0.54
**9977819**	-2.14	-0.93	0.53
**6419834**	-2.14	-0.93	0.53
67161540	-2.13	-0.94	0.52
**11485656**	-2.10	-0.87	0.54
16757867	-2.09	-0.73	0.68
**6711154**	-2.08	-0.93	0.68
10142586	-2.07	-0.99	0.52
657806	-2.07	-0.48	0.66
9532258	-2.05	-0.86	0.71
10209082	-2.01	-0.93	0.66
**24889392**	-2.00	-0.87	0.65
5782470	-1.99	-0.64	0.75
660914	-1.95	-0.22	0.67
1552034	-1.91	-0.50	0.79
3246585	-1.89	-0.55	0.66
5284352	-1.86	-0.48	0.62
16235522	-1.84	-0.42	0.71
5284416	-1.84	-0.72	0.65
661761	-1.83	-0.23	0.66
6097179	-1.82	-0.43	0.65
1745927	-1.82	-0.34	0.75
3246543	-1.82	-0.63	0.63
6918515	-1.81	0.31	0.75
5765289	-1.80	-0.47	0.78
664864	-1.78	-0.25	0.66
1363897	-1.78	-0.19	0.71
3246495	-1.78	-0.50	0.65
6604530	-1.78	-0.50	0.65
663169	-1.78	-0.50	0.65
456214	-1.78	-0.45	0.63
660368	-1.77	-0.40	0.65
660838	-1.77	-0.52	0.65

Commercially available compounds selected for the study are indicated in bold.

Supplementary Table 6. Complete list of compounds identified as potential hits from the *Stm* predictive model output.

PubChem ID	Predicted bacterial load z-score	Predicted cell viability z-score	Reliability
5035	-1.88	0.60	0.65
50994498	-1.68	0.52	0.72
202478	-1.58	0.47	0.71
**7333**	-1.56	0.04	0.66
44474938	-1.55	0.54	0.73
57402462	-1.55	0.54	0.73
11743300	-1.52	0.44	0.73
13998486	-1.52	0.44	0.73
15163141	-1.52	0.44	0.73
185834	-1.52	0.44	0.73
**4416**	-1.52	0.44	0.73
44303090	-1.52	0.44	0.73
44398003	-1.52	0.44	0.73
44398036	-1.52	0.44	0.73
44398114	-1.52	0.44	0.73
**47641**	-1.52	0.44	0.73
50266	-1.52	0.44	0.73
5474589	-1.52	0.44	0.73
6437849	-1.52	0.44	0.73
6439331	-1.52	0.44	0.73
65638	-1.52	0.44	0.73
6713949	-1.52	0.44	0.73
72027	-1.52	0.44	0.73
73345319	-1.52	0.44	0.73
93365	-1.52	0.44	0.73
9799239	-1.52	0.44	0.73
9841596	-1.52	0.44	0.73
9951886	-1.52	0.44	0.73
9954083	-1.52	0.44	0.73
**9417**	-1.51	0.15	0.68

Commercially available compounds selected for the study are indicated in bold.

Supplementary References

1. Kuijl, C. *et al.* Intracellular bacterial growth is controlled by a kinase network around PKB/AKT1. *Nature* **450,** 725–730 (2007).
2. Le Poole, I. C. *et al.* Phagocytosis by normal human melanocytes in vitro. *Exp. Cell Res.* **205,** 388–395 (1993).
3. Martínez-Lorenzo, M. J., Méresse, S., de Chastellier, C. & Gorvel, J. P. Unusual intracellular trafficking of Salmonella typhimurium in human melanoma cells. *Cellular Microbiology* **3,** 407–416 (2001).
4. Kaufmann, S. H. How can immunology contribute to the control of tuberculosis? *Nat Rev Immunol* **1,** 20–30 (2001).
5. Santos, R. L. *et al.* Animal models of Salmonella infections: enteritis versus typhoid fever. *Microbes Infect.* **3,** 1335–1344 (2001).
6. Ottenhoff, T. H. M. New pathways of protective and pathological host defense to mycobacteria. *Trends Microbiol.* **20,** 419–428 (2012).
7. Vergne, I., Chua, J., Singh, S. B. & Deretic, V. Cell biology of mycobacterium tuberculosis phagosome. *Annu. Rev. Cell Dev. Biol.* **20,** 367–394 (2004).
8. Verkhusha, V. V. *et al.* High stability of Discosoma DsRed as compared to Aequorea EGFP. *Biochemistry* **42,** 7879–7884 (2003).
9. Li, X. *et al.* Generation of destabilized green fluorescent protein as a transcription reporter. *J. Biol. Chem.* **273,** 34970–34975 (1998).
10. Basso, A. D. *et al.* Akt forms an intracellular complex with heat shock protein 90 (Hsp90) and Cdc37 and is destabilized by inhibitors of Hsp90 function. *J. Biol. Chem.* **277,** 39858–39866 (2002).
11. Liao, Y. *et al.* Peptidyl-prolyl cis/trans isomerase Pin1 is critical for the regulation of PKB/Akt stability and activation phosphorylation. *Oncogene* **28,** 2436–2445 (2009).
12. *Relational Data Mining*. (Springer Berlin Heidelberg, 2001). doi:10.1007/978-3-662-04599-2
13. Kocev, D., Vens, C., Struyf, J. & Džeroski, S. Tree ensembles for predicting structured outputs. *Pattern Recognition* (2013).
14. Blockeel, H., De Raedt, L. & Ramon, J. Top-down induction of clustering trees. *ICML Proceedings of the Fifteenth International Conference on Machine Learning* 55–63 (1998).
15. Breiman, L. Bagging predictors. *Machine learning* (1996).
16. Levatić, J., Ceci, M., Kocev, D. & Džeroski, S. Self-training for multi-target regression with tree ensembles. *Knowledge-Based Systems* (2017). doi:10.1016/j.knosys.2017.02.014
17. Gaora, P. O. Expression of genes in mycobacteria. *Methods Mol. Biol.* **101,** 261–273 (1998).
18. Coussens, N. P. *et al.* HTS Assay Validation. (2004).
